# Supplementary material for: Neuromolecular responses in disrupted mutualistic cleaning interactions under future environmental conditions
Source: BMC Biol. 2023 Nov 14;21:258. doi: 10.1186/s12915-023-01761-5 (PMC10644551; doi:10.1186/s12915-023-01761-5)
Supplement: Supplementary file 2 — Additional file 2: Figure S1. Unique and overlapping differentially expressed genes (DEGs) present in the warming condition for L. dimidiatus. Figure S2. Unique and overlapping differentially expressed genes (DEGs) present in the high CO2 condition for L. dimidiatus. Figure S3. Unique and overlapping differentially expressed genes (DEGs) present in the warming& high CO2 condition for L. dimidiatus. Figure S4. Unique and overlapping differentially expressed genes (DEGs) present in the warming condition for A. leucosternon. Figure S5. Unique and overlapping differentially expressed genes (DEGs) present in the high CO2 condition for A. leucosternon. Figure S6. Unique and overlapping differentially expressed genes (DEGs) present in warming & high CO2 condition for A. leucosternon. Figure S7.PCA’s of normalized gene counts using the design ~brain_region + treatment and a rlog transformation (rld) for each environmental treatment: a) warming, b) high CO2 and c) warming & high CO2) for A. leucosternon. Figure S8. PCA’s of normalized gene counts using the design ~brain_region + treatment and a rlog transformation (rld) for each environmental treatment: a) warming, b) high CO2 and c) warming & high CO2 for L, dimidiatus. [file 12915_2023_1761_MOESM2_ESM.docx]

Neuromolecular responses in disrupted mutualistic cleaning interactions under future environmental conditions

Ramírez-Calero, S. ^1,2^, Paula, J. R. ^3,4^, Otjacques, E. ^3,5,6^, Ravasi, T. ^7,8^,Rosa, R. ^3,4^, Schunter C.^1^*

*^1^The Swire Institute of Marine Science, School of Biological Sciences, The University of Hong Kong, Pokfulam Rd, Hong Kong SAR*

*^2^Departament de Biologia Marina, Institut de Ciències del Mar (CSIC*), Pg. Marítim de la Barceloneta 37-49, *Barcelona, Spain*

*^3^MARE – Marine and Environmental Sciences Centre & ARNET – Aquatic Research Network, Laboratório Marítimo da Guia, Faculdade de Ciências, Universidade de Lisboa, Av. Nossa Senhora do Cabo, 939, 2750-374 Cascais, Portugal*

*^4^Departamento de Biologia Animal, Faculdade de Ciências Universidade de Lisboa, Campo Grande, 1749-016, Lisbon, Portugal*

*^5^Carnegie Institution for Science, Division of Biosphere Sciences and Engineering, Church Laboratory, California Institute of Technology, 1200 E. California Blvd., Pasadena, CA 91125, USA*

*^6^ MARE – Marine and Environmental Sciences Centre & ARNET – Aquatic Research Network, University of Coimbra, Department of Life Sciences, 3000-456, Coimbra, Portugal*

*^7^Marine Climate Change Unit, Okinawa Institute of Science and Technology Graduate University, 1919–1 Tancha, Onna-son, Okinawa 904–0495, Japan*

*^8^Australian Research Council Centre of Excellence for Coral Reef Studies, James Cook University, Townsville, Queensland, 4811, Australia*

*(*) Correspondence: Celia Schunter,* [*celiaschunter@gmail.com*](mailto:celiaschunter@gmail.com)

Tel: +852 2299 0304

**SUPPLEMENTARY FIGURES**


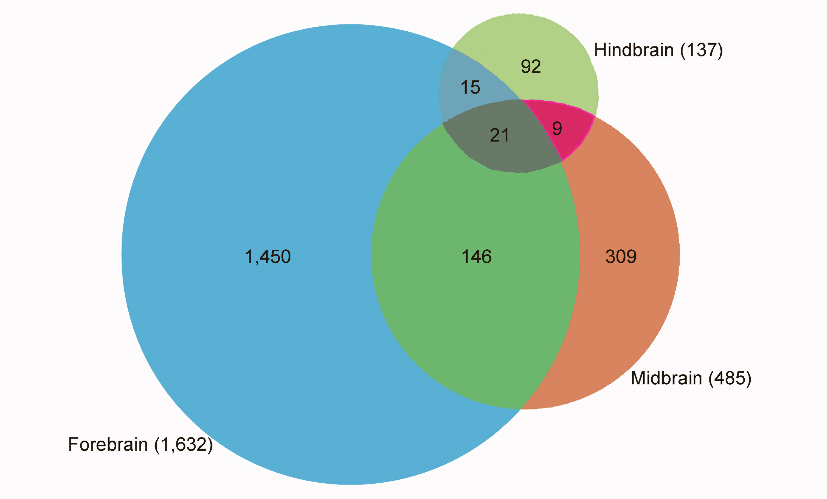


**Figure S1.** Unique and overlapping differentially expressed genes (DEGs) present in the Warming condition for *L. dimidiatus*


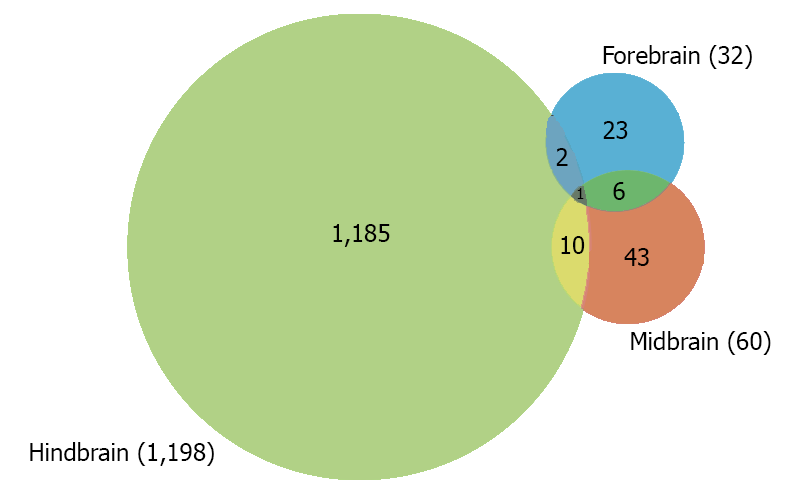


**Figure S2.** Unique and overlapping differentially expressed genes (DEGs) present in the High CO_2_ condition for *L. dimidiatus*


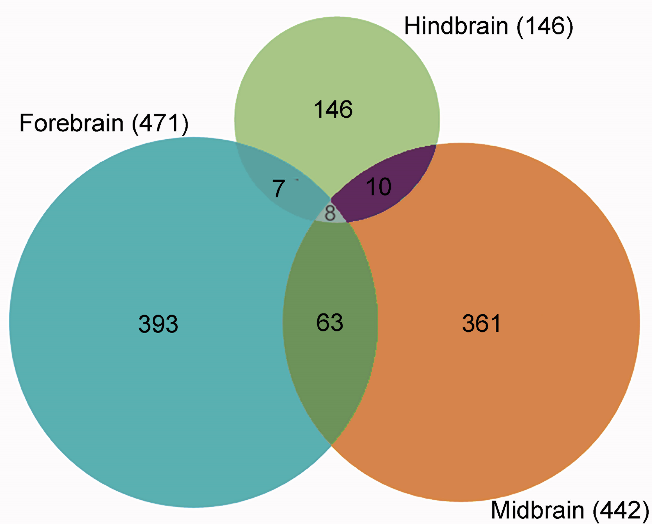


**Figure S3**. Unique and overlapping differentially expressed genes (DEGs) present in the Warming & High CO_2_ condition for *L. dimidiatus*.


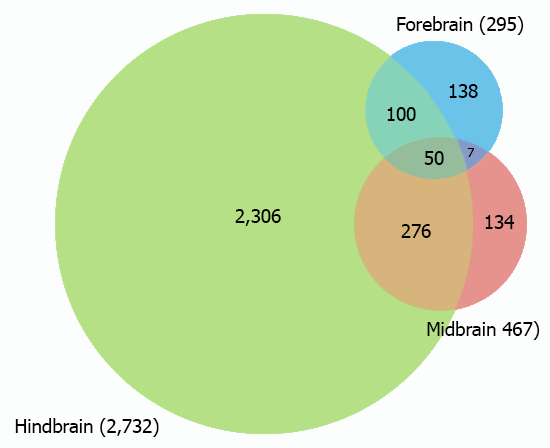


**Figure S4.** Unique and overlapping differentially expressed genes (DEGs) present in the Warming condition for *A. leucosternon.*


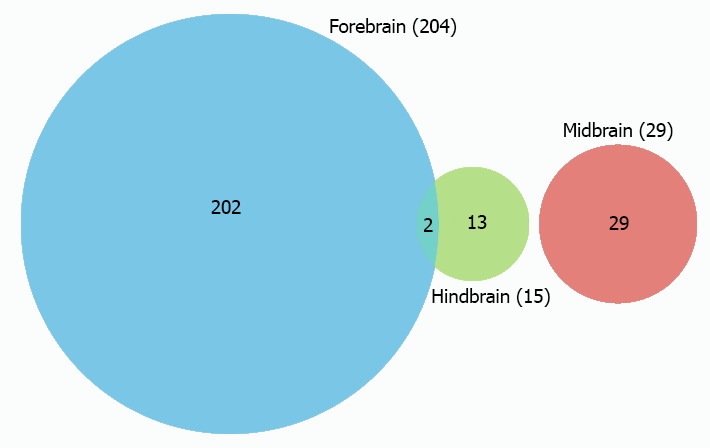


**Figure S5.** Unique and overlapping differentially expressed genes (DEGs) present in the High CO_2_ condition for *A. leucosternon.*


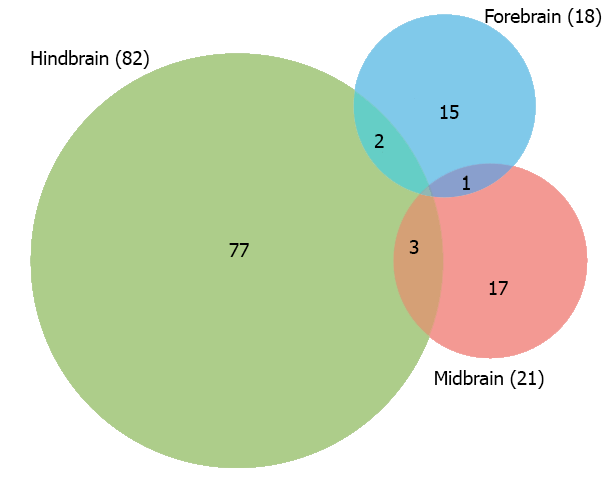


**Figure S6.** Unique and overlapping differentially expressed genes (DEGs) present in Warming & High CO_2_ condition for *A. leucosternon.*


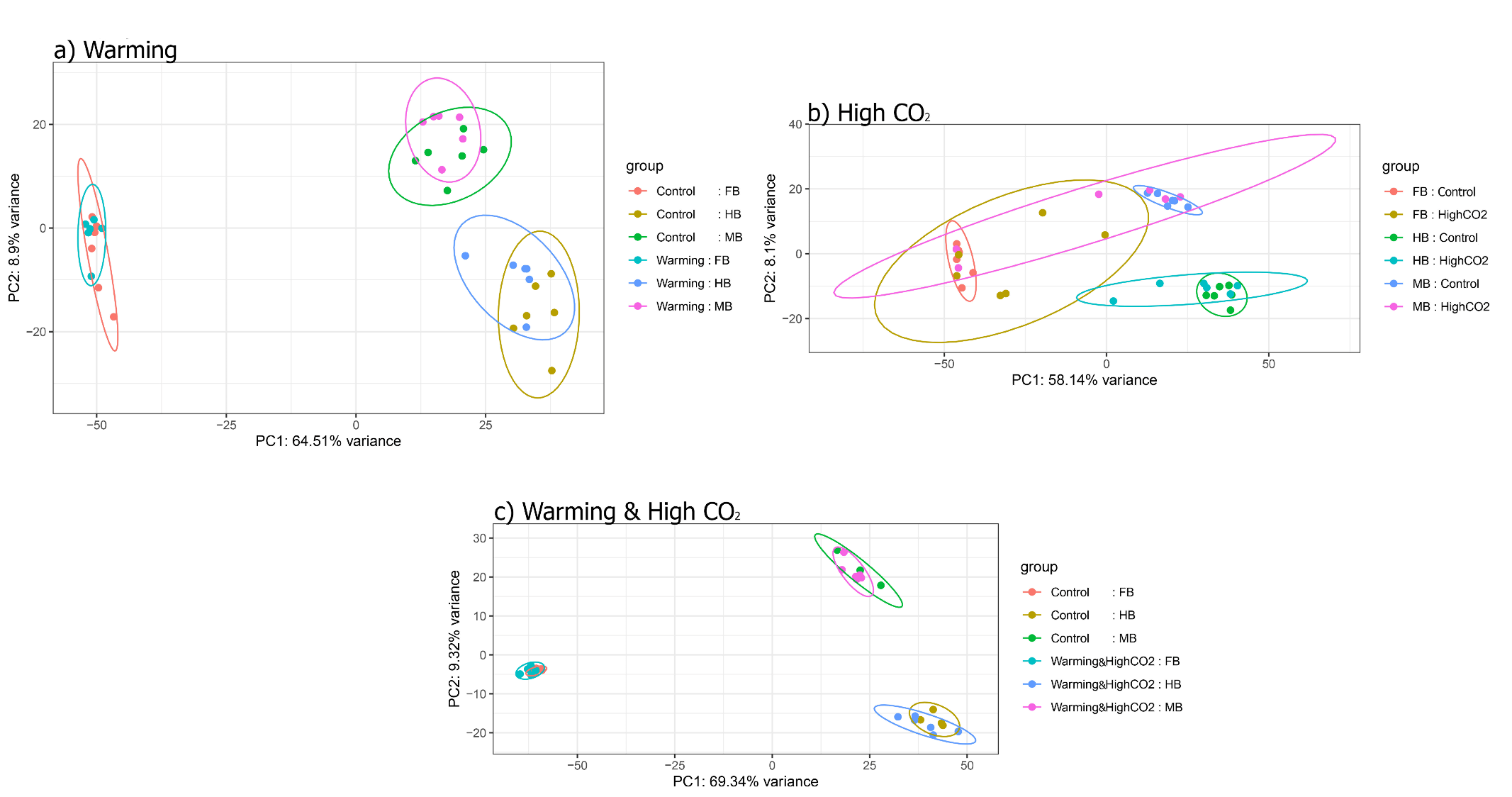


**Figure S7**. PCA’s of normalized gene counts using the design *~brain_region + treatment* and a *rlog* transformation (rld) for each environmental treatment: a) Warming, b) High CO_2_ and c) Warming & High CO_2_) for *A. leucosternon.*


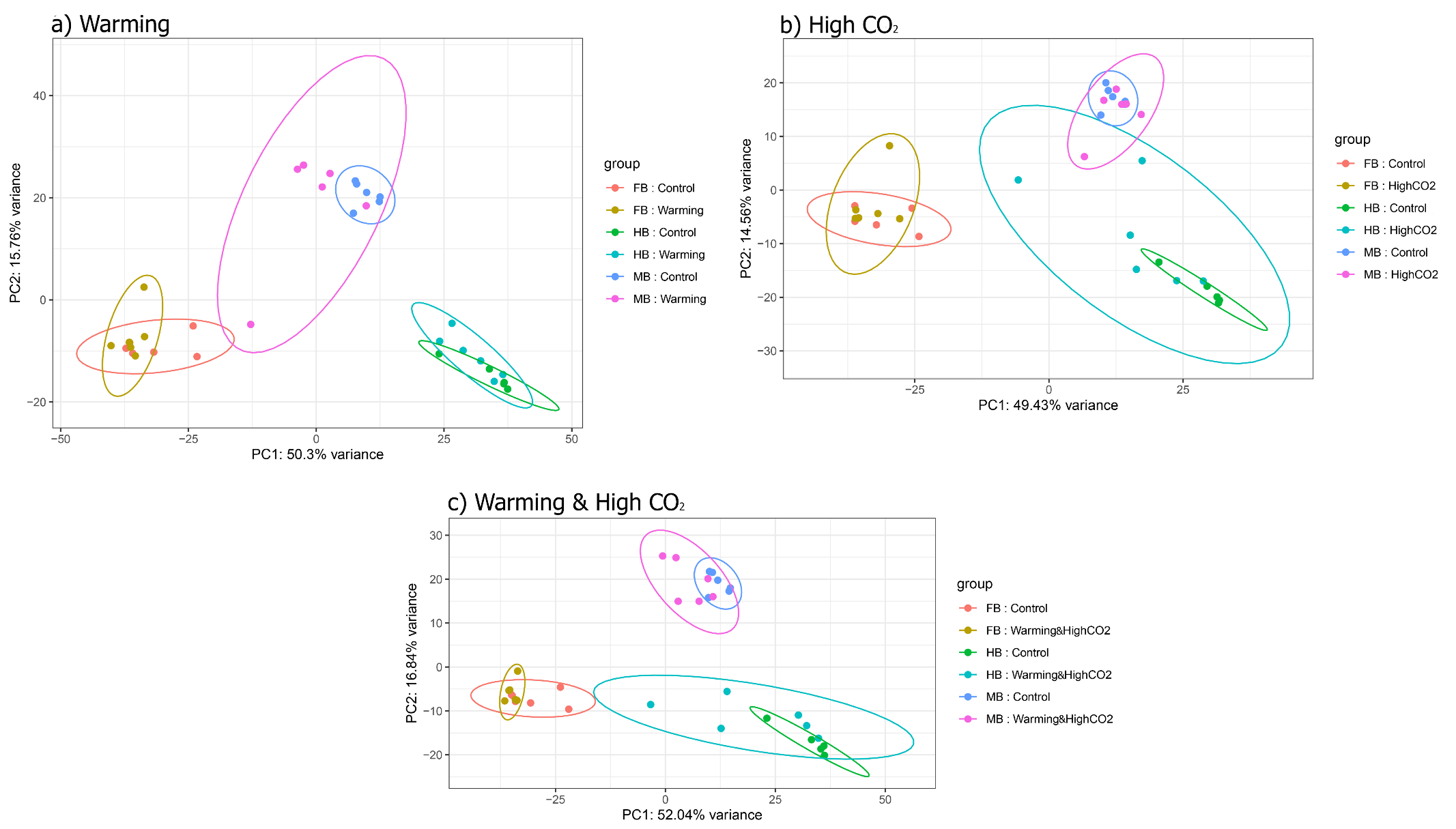


**Figure S8**. PCA’s of normalized gene counts using the design *~brain_region + treatment* and a *rlog* transformation (rld) for each environmental treatment: a) Warming, b) High CO_2_ and c) Warming & High CO_2_ for *L, dimidiatus*.
